# Watchman, What of the Night?

**Published:** 1888-05-12

**Authors:** 


### "WATCHMAN, WHAT OF THE NIGHT?"

(FOUNDED ON FACT.)

THE lights are low in the children's ward, and all the little ones, with the exception of wee Willie, are asleep. Poor little man, he keeps calling in his feeble voice for "Nursie! Nursie!" but no answer comes from Nurse, who is fast asleep, lying back in an easy chair by the fire, comfortably rolled up in a shawl. The night Sister has been round once, and would not be there again until early morning; Nurse will be up and doing before then, so is not likely to be caught napping. Ah! Nurse, sleep on while—no help being at hand—wee Willie bleeds to death; for God's messenger stole through the silent ward, and bore the little sufferer away to the Fatherland. Presently, in the chill of the early dawn, Nurse wakes with a shiver. She feels that something is amiss, looks nervously round the ward, and then steps over to the little cot where the small sufferer has lain so long. She starts—he looks so strange; and, she sees that from his mouth a stream of blood had poured. Alas! he will cry for Nurse no more, for the frail young life had ebbed away in that terrible stream! Nurse is stunned. She knows not what to do; she stands as one stupefied as she thinks that the Angel of Death has been in attendance while she slept at her post! What can she say to Sister? Some lie must be told. She hears her in the next ward. Oh dear! what can she do? What must she say? Before she can make up her mind Sister is by her side, gazing in horror at the sight before her: Nurse standing there with cap all awry, hair tumbled, apron all creased, and the child, that seemed so much better last night, a corpse. She, quick-sighted, takes all in at a glance, and is horrified. Nurse tries to speak, but Sister says quietly: "You had better go off duty. I will get Nurse Mary up to do the work and attend to this. Go; and may God help and forgive you."

There is no more to be told; the affair was hushed up, and the nurse discharged with a severe reprimand. Let us hope there are none like her in our hospitals at the present day. If this little story falls into the hands of any one who is tempted when on night duty to sleep at her post, let her remember what *might* happen if she relaxes her vigilance even for a moment. S. ROSE MACBRIDE.

